# *Fusobacterium nucleatum* promotes liver metastasis in colorectal cancer by regulating the hepatic immune niche and altering gut microbiota

**DOI:** 10.18632/aging.203914

**Published:** 2022-02-25

**Authors:** Han Yin, Zhuangzhuang Miao, Lu Wang, Beibei Su, Chaofan Liu, Yu Jin, Bili Wu, Hu Han, Xianglin Yuan

**Affiliations:** 1 Department of Oncology, Tongji Hospital, Huazhong University of Science and Technology, Wuhan, China; 2 Department of Neurosurgery, Tongji Hospital, Huazhong University of Science and Technology, Wuhan, China

**Keywords:** *Fusobacterium nucleatum*, colorectal cancer, metastasis, immune microenvironment, gut microbiota

## Abstract

Liver metastasis is the major cause of death in colorectal cancer (CRC) patients. Nevertheless, the underlying mechanisms remain unknown. Gut microbiota intricately affect the initiation and progression of CRC by instigating immune response through the secretion of pro-inflammatory cytokines. In this study, we investigated the contribution of *Fusobacterium nucleatum (F.nucleatum)* to the microbiota-liver axis of CRC in mice, focusing on the correlation between liver immunity and gut microbiota alterations. When *F. nucleatum* was orally administered to mice, CRC liver metastasis was evidently exaggerated and accompanied by noticeable deleterious effects on body weight, cecum weight, and overall survival time. Further evaluation of the immune response and cytokine profiles revealed a substantial increase in the levels of pro-inflammatory cytokines such as IL6, IL12, IL9, IL17A, CXCL1, MCP-1, TNF-α, and IFN-γ in the plasma of mice treated with *F. nucleatum* as compared to that in the untreated control mice. Besides, hepatic immune response was also modulated by recruitment of myeloid-derived suppressor cells, reduction in the infiltration of natural killer (NK) and T helper-17 (Th17) cells, as well as increase in regulatory T cell accumulation in the liver. Additionally, sustained *F. nucleatum* exposure abridged the murine gut microbiota diversity, inducing an imbalanced and restructured intestinal microflora. In particular, the abundance of CRC-promoting bacteria such as *Enterococcus* and *Escherichia/Shigella* was evidently elevated post *F. nucleatum* treatment*.* Thus, our findings suggest that *F. nucleatum* might be an important factor involved in promoting CRC liver metastasis by triggering of liver immunity through the regulation of gut microbiota structure and composition.

## INTRODUCTION

CRC is the third-leading cause of cancer-related deaths worldwide [1]. More than 50 % of patients progress to metastasis during the course of this disease, making it the leading cause of CRC-associated deaths [2]. Liver is the primary target organ for distant metastasis in CRC patients. Approximately 25 % of patients present with synchronous liver metastases at initial diagnosis [3]. However, only less than 15 % of patients with CRC liver metastasis (CRLM) meet the criteria for surgical resection. Nevertheless, even after surgery, relapses generally occur in 75 % of patients, with approximately half of them occurring in the liver leading to high mortality in CRC patients [4]. Although chemotherapy and targeted medicines for unresectable CRLM have made tremendous advances for decades, its prognosis remains poor [5, 6]. Hence, elucidating the underlying mechanism of CRLM will be beneficial for the development of efficacious novel CRC therapeutics.

The gut commensal microbiota is a complex system that regulates the host immunity. Modulation of intestinal microbiota composition has been documented to contribute toward the disruption of the ecosystem, favoring abnormal immune activation to support or prevent tumor growth [7]. In fact, both specific bacteria and dysbacteriosis have indicated a complex link with colorectal carcinogenesis through the activation of immunity [8, 9]. Dysregulation of gut microbiota has been reported to instigate a pro-inflammatory microenvironment by regulating the cytokine activity and its interaction with the immune system, thereby promoting tumor growth [10].

In terms of CRLM, the liver is exposed to gut metabolites and components through the portal vein and enterohepatic circulation, which is intensely affected by gut microbiota in hepatocellular cancer [11] and hepatic metastasis [12]. Numerous studies have demonstrated that the gut microbiota mediates the secretion of inflammatory cytokines such as IL6, IL12, IL17, tumor necrosis factor-α (TNF-α), and interferon-γ (IFN-γ) to form an immune suppressive microenvironment in the liver to facilitate CRLM progression [13, 14]. Moreover, gut microbiota modulate the accumulation of hepatic natural killer T(NKT) cells in a chemokine (C-X-C motif) ligand 16–dependent manner by affecting bile acid metabolism [14]. Sodium butyrate is also found to regulate the gut microbiome and the balance between regulatory T (Treg) and Th17 cells in the murine liver [13]. In particular, studies have reported that *F. nucleatum* enhances CRC invasion, proliferation, and recurrence by activating pro-inflammatory pathways [15, 16], triggering mucosa-associated inflammation [17] and blocking the cytotoxic anti-tumor activity of T cells [18] and NK cells [19]. Furthermore, it has been evidently shown that *F. nucleatum* can be detected in liver metastases by fluorescence *in situ* hybridization and that antibiotics can inhibit the growth of *F. nucleatum-*infected tumors, suggesting that *F. nucleatum* facilitates cancer cell migration and proliferation in CRC [20].

Nevertheless, the effects of *F. nucleatum* on the immune response in relation to inflammatory cytokines in the liver, as well as the changes in the structure and composition of gut microbiota have not been comprehensively investigated. Hence, this study aimed to evaluate the influence of *F. nucleatum* on the diversity and composition of gut microbiome using a murine model. Further, the effect of *F. nucleatum–*mediated gut microbiome alterations on the innate and adaptive immune response leading to augmented hepatic metastases were also investigated, thus providing potent novel insights for developing an effective treatment strategy for CRLM.

## RESULTS

### Administration of *F. nucleatum* promotes liver metastasis of CRC in mice

**Figure 1:**
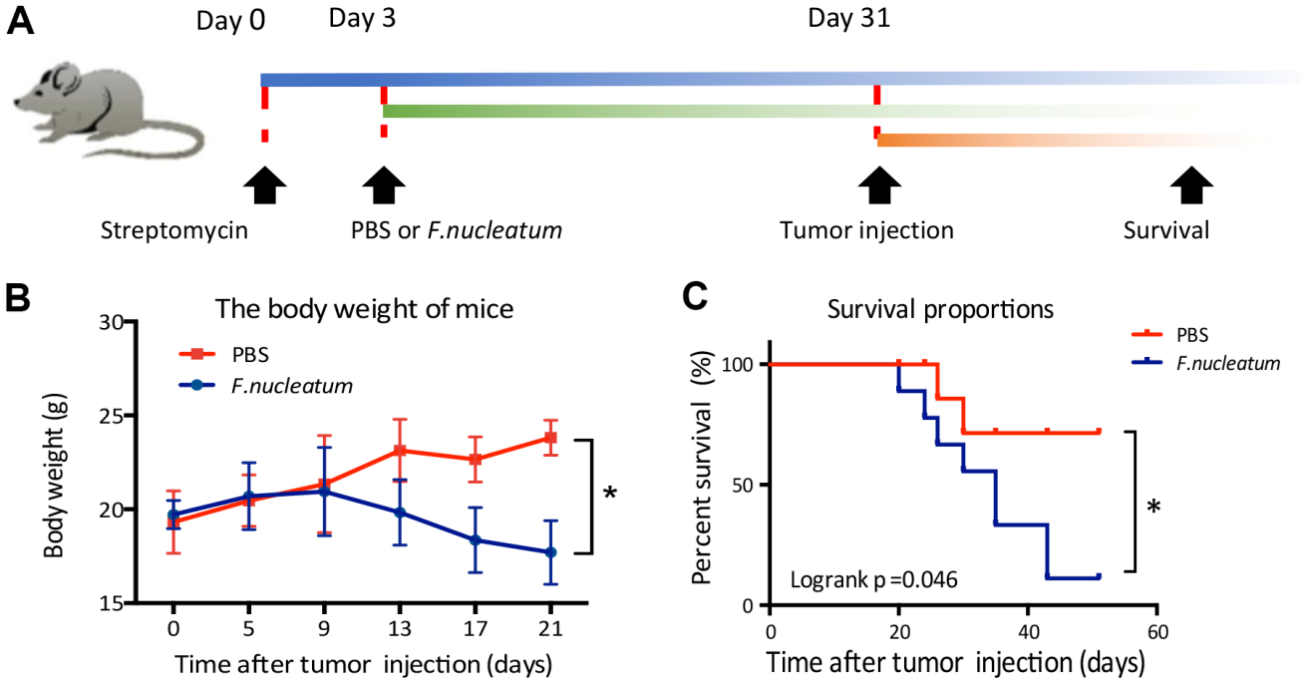
***Fusobacterium nucleatum* affects the weight and survival time of mice in a model of liver metastasis of colorectal cancer.** (**A**) Schematic of the experimental setup. (**B**, **C**) The effects of *F. nucleatum* administration on body weight (**B**) and survival time (**C**) (n=15 per group).

To investigate the effect of *F. nucleatum* on the phenomenon of CRLM, 1×10^6^ CT26-Luc cells were injected beneath the splenic capsule of mice to establish a liver metastasis model after a 4-week administration of either *F. nucleatum* or PBS (Figure 1A). Subsequent analyses revealed that the mice treated with *F. nucleatum* demonstrated significant weight loss at the second week post tumor injection in addition to a median survival time of less than 40 days as compared to the median survival time of 60 days in mice treated with PBS (Figure 1B, 1C).

**Figure 2:**
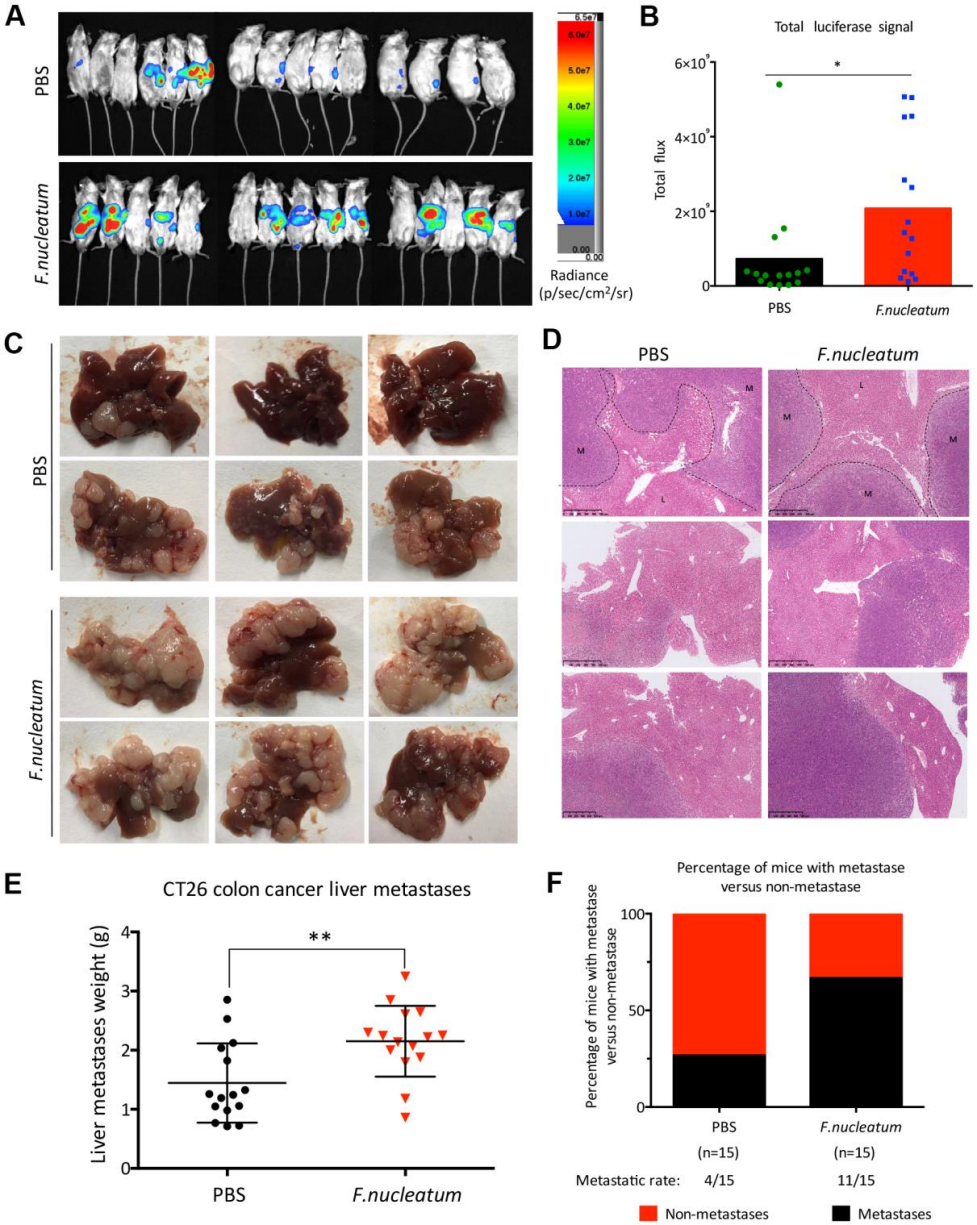
**Administration of *Fusobacterium nucleatum* promotes liver metastasis of colorectal cancer in mice.** (**A**, **B**) PBS-resuspended 10^9^ colony-forming units of *F. nucleatum* or PBS were administrated to mice for 8 weeks by gavage every day. The mice received intra-splenic injection post 2-week administration of *F. nucleatum* or PBS intragastrically. At day 28 after intra-splenic transplantation of CT26-Luc cells, *in vivo* bioluminescence imaging was used to assess liver metastases (**A**) of BALB/c mice (n=15 per group). (**B**) The corresponding data expressed by the change in the total signal flux. Data are presented as mean ± standard deviation (SD; *P < 0.05, **P < 0.01, and ***P < 0.001; unpaired Student’s t-test). (**C**, **D**) After the 2-week treatment of *F. nucleatum* or PBS, mice received intra-splenic injection of CT26 tumor cells. Four weeks later, the mice were euthanized, and subsequent histological studies were performed. The representative metastatic foci (**C**) and hematoxylin and eosin-stained liver sections (**D**) in mice are shown. (**E**, **F**) Livers were weighed (**E**) and liver metastatic rates were measured (**F**). Data are presented as mean ± SD (*P < 0.05, **P < 0.01, and ***P < 0.001; unpaired Student’s t-test).

To further determine whether *F. nucleatum* infection promotes CRLM, we used an *in vivo* optical imaging system to monitor the liver metastases of CT26-Luc cells using a murine CRC model. Our results showed that on the 28^th^ day post establishment of the model, CT26-Luc cells were found to be successfully seeded in the spleens of mice (Figure 2A). Furthermore, the liver metastatic rate in PBS-treated mice (n = 4) and *F. nucleatum*–treated mice (n = 10) were determined to be 26.67 % and 66.67 % respectively (Figure 2F). Subsequent region of interest (ROI) analysis of the optical *in vivo* imaging studies showed that the mean relative luminescence count (number of photons emitted per second) in the livers of *F. nucleatum*–treated mice was significantly higher than that in the PBS-treated mice (Figure 2B). Moreover, enhanced metastases and a higher tumor burden were observed in mice after *F. nucleatum* treatment as compared to that in PBS-treated mice (Figure 2C, 2D). Particularly, the metastatic liver weight of *F. nucleatum*–treated mice was determined to be markedly higher than that of the control group (Figure 2E).

### *F. nucleatum* augments the levels of inflammatory cytokines in plasma

**Figure 3:**
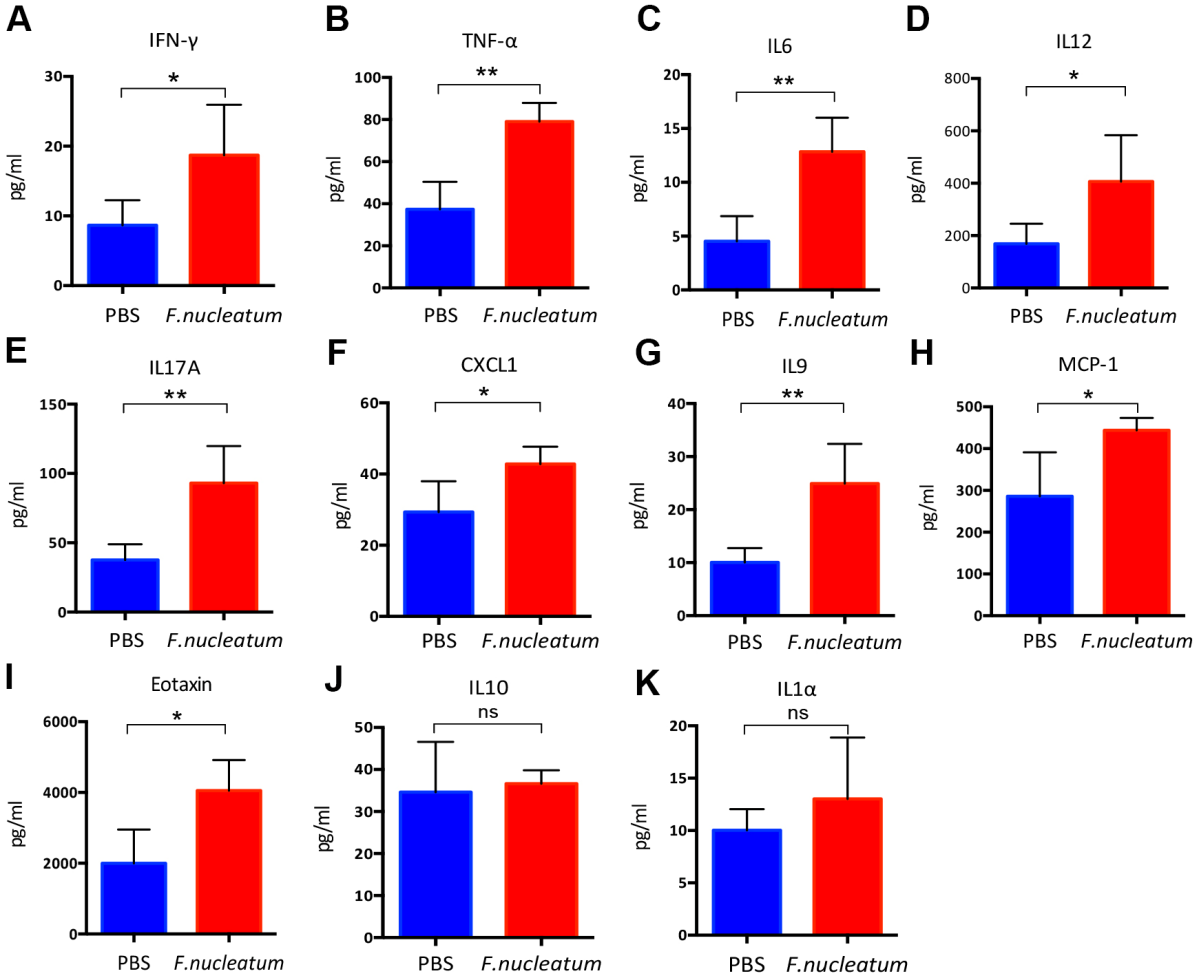
***Fusobacterium nucleatum* regulates the levels of inflammatory cytokines in mice plasma.** (**A**–**K**) Concentrations of the pro-inflammatory cytokines, (**A**) Interferon-gamma (IFN-γ), (**B**) tumor necrosis factor-α (TNF-α), (**C**) IL6, (**D**) IL12, (**E**) IL17A, (**F**) chemokine (C-X-C) motif ligand (CXCL1), (**G**) IL9, (**H**) macrophage chemoattractant protein-1(MCP-1), (**I**) Eotaxin, (**J**) IL10, (**K**) IL1α in murine plasma were detected by the Bio-Plex Pro Mouse Cytokine Array kit. Data are presented as mean ± standard deviation (n=5 per group). *P < 0.05, **P < 0.01, and ***P < 0.001; unpaired Student’s t-test.

To determine the effect of *F. nucleatum* administration on the levels of inflammatory cytokines in the plasma of mice, a cytokine antibody array was employed. The results revealed that the plasma from the *F. nucleatum* group exhibited higher levels of pro-inflammatory cytokines as compared to that from the PBS-administered group. Previous studies have illustrated that *Fusobacterium-*enriched adenoma cases exhibit increased secretion of inflammatory cytokines [21]. Consistent with this finding, our study also demonstrated that the *F. nucleatum–*treated mice exhibit significantly increased levels of the pro-inflammatory cytokines such as IFN-γ, TNF-α, IL6, IL12, IL17A, chemokine (C-X-C motif) ligand 1 (CXCL1), IL9, macrophage chemoattractant protein-1(MCP-1), and Eotaxin as compared to the PBS-treated mice (Figure 3A–3I). In contrast, there were no significant changes in the secretion of IL10 and IL1α (Figure 3J, 3K).

### *F. nucleatum* aggravates CRLM by remodeling the liver immune microenvironment

**Figure 4:**
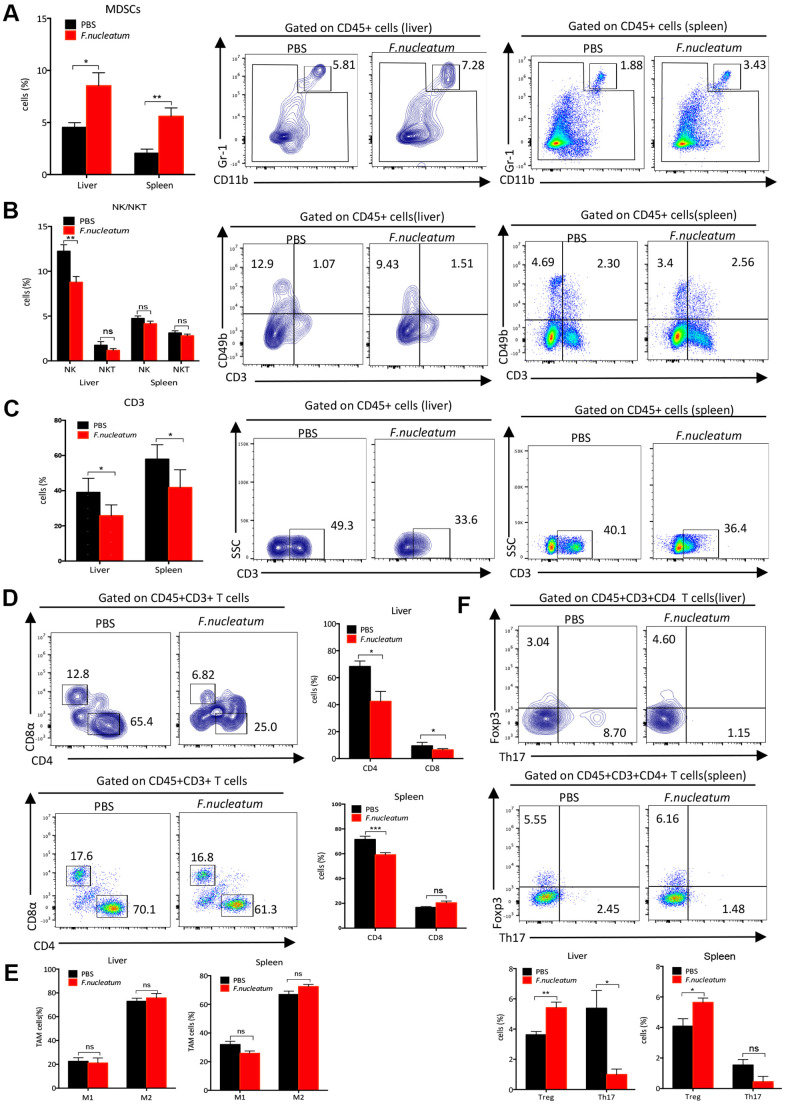
***Fusobacterium nucleatum* aggravates colorectal cancer liver metastasis by restructuring the liver immune microenvironment.** (**A**) Myeloid-derived suppressor cells (MDSCs; CD45^+^CD11b^+^Gr-1^+^) in the liver and spleen were determined by flow cytometry. (**B**) NK (CD45^+^CD3^-^CD49b^+^) and NKT cells (CD45^+^CD3^+^CD49b^+^) were analyzed by flow cytometry. (**C**, **D**) CD3^+^T cells (**C**), CD3^+^CD4^+^T cells and CD3^+^CD8^+^T cells (**D**) in the liver and spleen were assessed by flow cytometry and analyzed by gating on lymphocytes and CD3^+^ cells. (**E**) Tumor-associated macrophages (CD45^+^CD11b^+^F4/80^+^) in the liver and spleen were determined by flow cytometry. (**F**) Treg cells (CD4^+^Foxp3^+^) and Th17 cells (IL17^+^CD4^+^) were evaluated by flow cytometry and analyzed by gating on CD4^+^T cells. Data are presented as mean ± standard deviation (n=5 per group). *P < 0.05, **P < 0.01, and ***P < 0.001; unpaired Student’s t-test.

To explore the immune mechanisms of tumor progression, we further evaluated the impact of *F. nucleatum* on the liver immune microenvironment. The analyses of the murine livers and spleens post treatment with PBS or *F. nucleatum* revealed that *F. nucleatum–*treated mice exhibit elevated peripheral and hepatic myeloid-derived suppressor cell (MDSC) (CD11b^+^Gr-1^+^) infiltration (Figure 4A), but decreased NK (CD3^-^CD49b^+^) as well as CD3^+^, CD4^+^, and CD8^+^ T cells in their livers as compared to the PBS-administered control mice (Figure 4B–4D). However, *F. nucleatum* exposure had no effect on NKT cells (CD3^+^CD49b^+^) (Figure 4B) or tumor-associated macrophages (TAM) (CD11b^+^F4/80^+^) (Figure 4E) in either the spleen or liver as compared to the PBS treatment of mice. Furthermore, the reduction of NK cells (Figure 4B) and CD8^+^ T cells (Figure 4D) was not detected in the spleens of *F. nucleatum*–treated mice, suggesting a liver-specific effect.

The Treg/Th17 cells balance mediates the pro- and anti-tumor immunity of CRC. To investigate whether *F. nucleatum* can modulate the tumor microenvironment (TME), Tregs (CD4^+^Foxp3^+^) and Th17 (IL17^+^CD4^+^) cells were detected in murine livers and spleens post establishment of CRC model in the *F. nucleatum* or PBS-administered mice. The results revealed that Treg lymphocytes were significantly elevated in the *F. nucleatum* group as compared to that in the PBS-administered group (Figure 4F), which corroborates with the tumor promoting role of Tregs in CRC progression [22]. Contrastingly, the Th17 cells were found to be reduced in livers but not spleen of mice after *F. nucleatum* administration in the liver as compared to those administered with PBS as control (Figure 4F). These results demonstrated that *F. nucleatum* plays a potent role in regulating the abundance of Tregs and Th17 cells to modulate anti-tumor immunity in murine livers.

### *F. nucleatum*–induced intestinal microbial dysbiosis is involved in the development of CRLM

**Figure 5:**
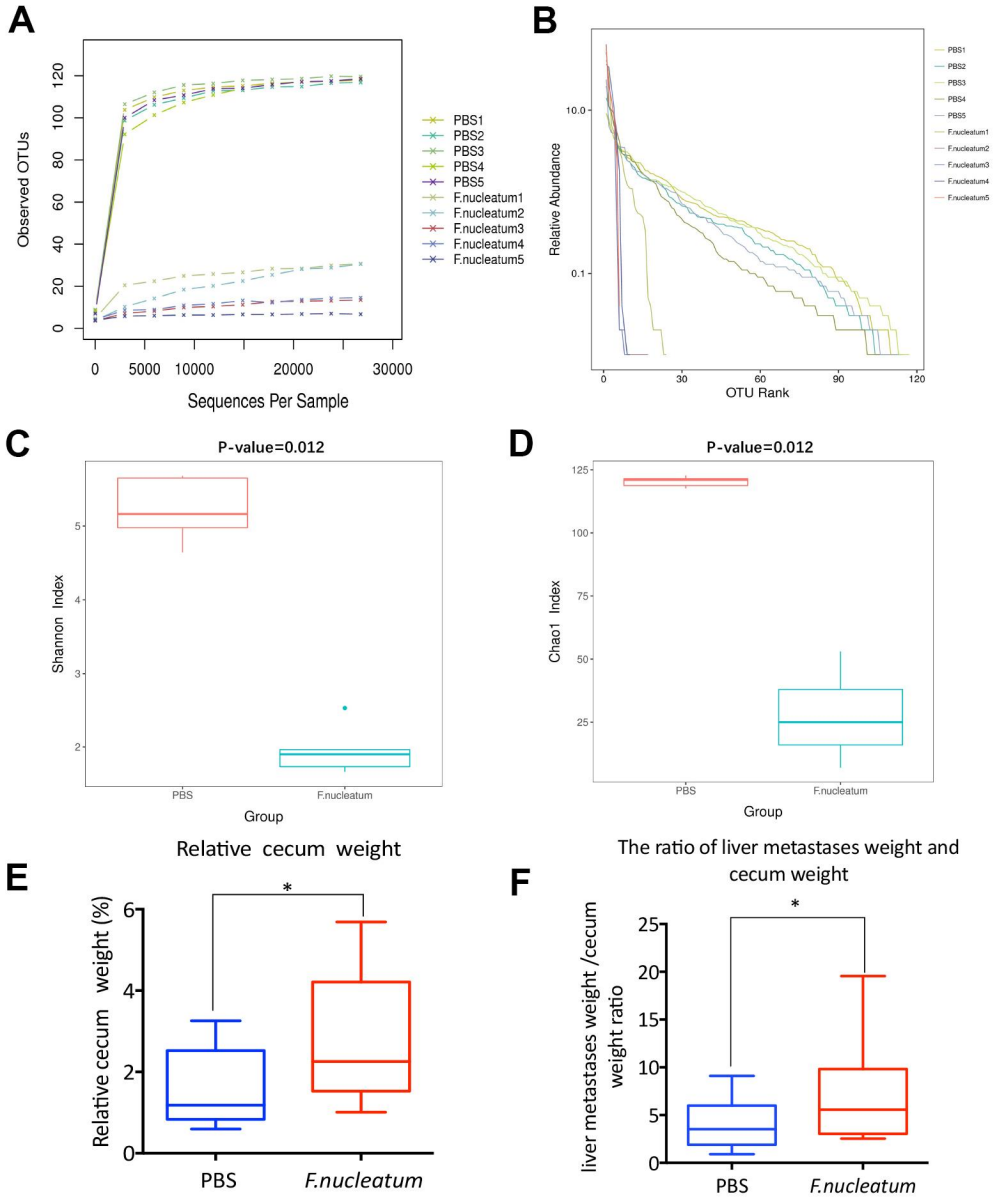
***Fusobacterium nucleatum–*induced intestinal microbial dysbiosis is involved in the development of colorectal cancer liver metastasis.** (**A**–**D**) The rarefaction curve (**A**), relative abundance curve (**B**), Shannon diversity index (**C**), and Chao1 diversity index (**D**) of intestinal bacteria in mice treated with *F. nucleatum* or PBS were assessed by 16S rRNA sequencing (n=5 per group). Data are presented as mean ± standard deviation (SD; unpaired Student’s t-test). (**E**, **F**) The cecum and livers of mice were dissociated and weighed. The relative cecum weight (cecum weight/body weight) (**E**) and the metastatic liver weight/cecum weight (MLW/CW) ratio (**F**) were measured. Data are presented as mean ± SD (n=15 per group). *P < 0.05, **P < 0.01, and ***P < 0.001; unpaired Student’s t-test.

To investigate the influence of gut microbiota changes related to *F. nucleatum* administration on the manifestation of CRLM, we aseptically collected stool samples from the distal rectum, immediately after euthanizing mice with established CRC treated with either *F. nucleatum* or PBS, for 16S rRNA gene sequencing. Subsequent analyses of the rarefaction curves illustrated that the species richness in the gut microbiome of the *F. nucleatum*–treated mice was evidently lower than that of PBS-treated mice (Figure 5A). Correspondingly, the abundance curve also showed reduced species richness and lower species uniformity in the gut microbiome of the *F. nucleatum–*treated mice (Figure 5B) as compared to that in the PBS-treated mice. Further evaluation of the microbial diversity using the Shannon Index (Figure 5C) and Chao1 Index (Figure 5D) revealed a significant reduction in species diversity upon *F. nucleatum* gavage as compared to that in control group. Overall, these findings suggest that *F. nucleatum* exerts a significant effect on the abundance and diversity of gut bacteria.

Previous studies have shown that dysbiosis and associated impairment in the gut microenvironment especially manifest in the distal gut, particularly in the cecum [23, 24]. Moreover, cecal weight has been reported to be associated with intestinal microbial abundance and diversity [25]. Consequently, the murine cecum as well as metastatic livers from the *F. nucleatum* or PBS-treated mice with established CRC were dissected post euthanization and weighed. The results revealed that *F. nucleatum–*treated mice demonstrated a higher relative cecum weight (cecum weight/body weight) (Figure 5E) and MLW/CW (metastatic liver weight/cecum weight) ratio (Figure 5F) than those noted with PBS-administered mice; which was concomitant with the increased tumor burden.

### Gut bacterial composition and community structure is altered post *F. nucleatum* administration

**Figure 6:**
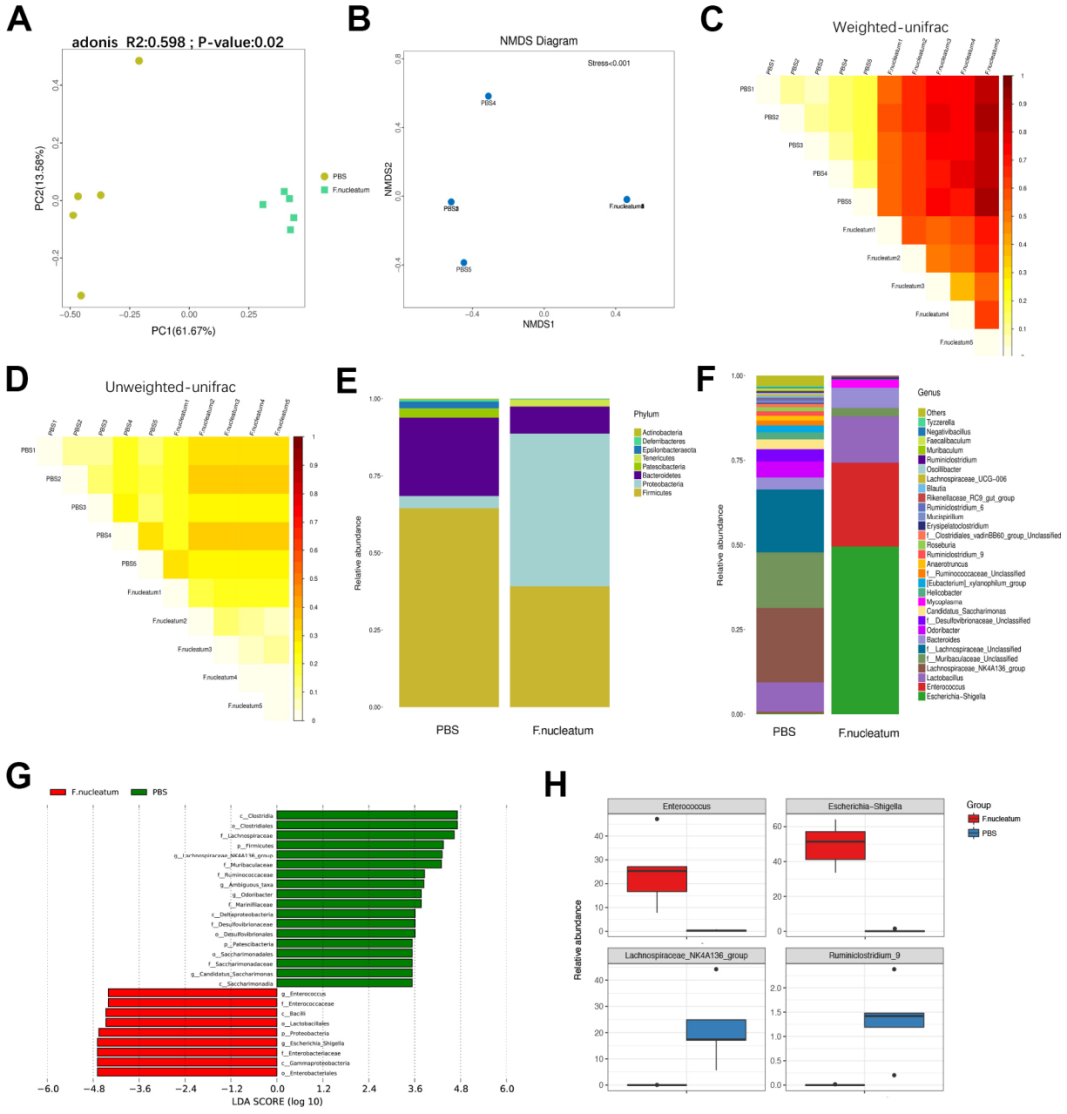
Gut bacterial composition and community structure is altered after *Fusobacterium nucleatum* administration (**A**) Principal component analysis was performed on 16S rRNA sequencing of stool samples from mice treated with either *F. nucleatum* or PBS (n=5 per group). Significant differences (*P*< 0.05) in intestinal microbiota composition between the two groups were analyzed via PERMANOVA (adonis). (**B**–**D**) The beta diversity of intestinal bacteria was compared by non-metric multidimensional scaling analysis (**B**) and weighted (**C**) and unweighted (**D**) UniFrac distance analysis between PBS- and *F. nucleatum–*treated groups. (**E**, **F**) The alteration of intestinal bacterial patterns at the phylum level (**E**) and genus level (**F**) in mouse stool from the PBS and *F. nucleatum* groups was assessed via 16S rRNA sequencing (n=5 per group). (**G**) Specific phylotypes of gut bacteria in the two groups using LEfSe. The histogram shows the LDA scores > 3.5 at the operational taxonomic unit level. (**H**) The top four genera with the largest difference between the two groups of mice treated with either *F. nucleatum* or PBS.

To determine the alteration in the gut bacterial composition and community structure upon treatment *F. nucleatum*, the fecal microbiota composition and community of mice treated with *F. nucleatum* or PBS were examined. The principal co-ordinate analysis (PCoA) and non-metric multidimensional scaling (NMDS) analysis revealed that the intestinal bacterial flora is substantially altered after *F. nucleatum* exposure as compared to that with PBS treatment (Figure 6A, 6B). The analysis of weighted and unweighted UniFrac distance further indicated that *F. nucleatum* instigated a disturbance in the microbiota, and that the gut bacterial community structure was significantly different between the *F. nucleatum* and PBS-administered groups (Figure 6C, 6D).

Furthermore*,* our results revealed that *F. nucleatum* gavage increases the relative abundance of *Proteobacteria* but decreases the relative abundance of Bacteroidetes at the phylum level (Figure 6E) as compared to PBS administration. The relative abundance of *Lachnospiraceae_NK4A136_group* was lower, but *Bacteroides* and *Lactobacillus* were significantly more abundant in the *F. nucleatum–*treated mice as compared to the PBS-administered mice (Figure 6F). Subsequent analysis using the linear discriminant analysis (LDA) effect size (LEfSe) algorithm indicated that the two groups had completely different taxa (Figure 6G). However, the specific groups with the largest difference between the two groups of mice were determined at the genus level (Figure 6H). Specifically, the *F. nucleatum–*treated mice contained a higher abundance of *Enterococcus* and *Escherichia/Shigella* as compared to PBS-treated mice. Moreover, the relative abundances of *Lachnospiraceae_NK4A136_group* and *Ruminiclostridium_9* were lower in *F. nucleatum–*treated as compared to the PBS-treated mice.

## DISCUSSION

Advances in high-throughput sequencing technology have enabled researchers to appreciate the crucial role of gut microbiota in the initiation and progression of CRC [26]. Studies have suggested a strong association of *F. nucleatum* enrichment in gut microbiota with colorectal carcinogenesis [27, 28] that potentiates CRC cell proliferation, adhesion, and invasion by activating relevant cancer-signaling pathways [15, 16, 29] or modulating the immune response [30, 31]. However, the potential role of *F. nucleatum* in CRLM remains poorly understood. In this study, we have elucidated that *F. nucleatum* administration promotes CRLM and is correlated with the activation of hepatic immune microenvironment. Interestingly, a recent study has innovatively demonstrated that *F. nucleatum* can be detected in liver metastases and the *F. nucleatum*-positive CRLM are associated with the reduced density of T cells [32]. Consistent with these findings, using *in vivo* murine models of CRC, we have shown that *F. nucleatum* reshapes the immune microenvironment in metastatic livers. Furthermore, our results also evidently revealed the crosstalk between *F. nucleatum*–induced dysbiosis and liver immune environment.

Our results confirmed that *F. nucleatum* modulates the immune response and promotes CRLM by affecting the gut microbiota using *in vivo* mice models of CRC. In this study, we have demonstrated that *F. nucleatum* administration significantly alters the plasma concentrations of different pro-inflammatory cytokines. Particularly, significant upsurges were observed in IL17A, TNF-α, IL6, IL12, Eotaxin, CXCL1, IFN-γ, MCP-1, and IL9 in the plasma concentrations of mice with established CRC upon administration of *F. nucleatum* as compared to that upon PBS treatment, suggesting an inflammation promoting role of *F. nucleatum*. Consistent with our findings, *F. nucleatum* has been documented to be enriched in inflammatory bowel disease (IBD) and colorectal adenomas [21], whereas in CRC, patients demonstrate higher concentrations of inflammatory cytokines as compared to healthy individuals [33–35].

The elevation in the levels of the pro-inflammatory cytokines such as MCP-1, Eotaxin, IL12, and IL6 is indicative of chronic inflammation. Several studies have proven the association of pro-inflammatory cytokines in the TME with tumor progression due to their ability to recruit CD8^+^ T cells, stimulate TAM polarization, accumulate Treg cells, and facilitate angiogenesis [36–40]. Accordingly, our results further corroborated that high levels of these cytokines are associated with CRC metastasis [41].

IFN-γ and IL9 play important roles in anti-tumor activity [42, 43]. However, IFN-γ can promote CRC proliferation and metastasis by inducing a pro-inflammatory effect [44], which is in agreement with our findings. In addition, IL9 expression in CRC samples has been reported to be significantly higher than that in the surrounding healthy mucosa, suggesting its tumorigenic role in CRC [45].

IL17A and TNF-α are important inflammatory cytokines linked to host immunity for CRC initiation, promotion, angiogenesis, and metastasis. IL17A promotes tumor progression by suppressing anti-tumor immune activity, including its impact on CD8^+^ T cell polarization to lose their cytotoxic ability [46, 47]. The higher concentrations of IL17A and TNF-α following *F. nucleatum* administration in our findings are therefore consistent with previous findings [48].

Our studies further revealed that *F. nucleatum* stimulation increases the number of MDSCs in the spleen and liver of mice with established CRC as compared to PBS-administered mice. The recruitment of MDSCs into the liver has been documented to modulate immunosuppression by inhibiting NK cell cytotoxicity and inducing the accumulation of Tregs [49]. Circulating MDSCs have been reported to correlate with tumor metastasis, clinical stage, and target treatment in CRC. Moreover, CXCL1 is critical for the formation of a pre-metastatic niche for CRC metastasis via the recruitment of MDSCs [50]. Thus, our findings showing higher levels of CXCL1 upon stimulation of mice with CRC by *F. nucleatum* indicate that *F. nucleatum* may be involved in the promotion of CRC metastasis, which is in agreement with a previous study [39].

NK cells are an abundant class of lymphocytes with an effective cytotoxic response and cytokine production that facilitate elimination of cancer. Primarily, these cells are vital effectors of the innate immune response that protect the host organism against pathogens via non-specific mechanisms [51]. In agreement with previous studies, our study has also shown that *F. nucleatum* treatment abolishes the innate immune response [52]. Furthermore, Gur et al. [19] have reported that the reduction of NK cells upon *F. nucleatum* treatment results in impaired NK cell–mediated cytotoxic activity in CRC tumors.

Further evaluation of peripheral and hepatic T cell responses in a murine CRC model administered with. *F. nucleatum* led to a decreased CD3^+^ T cell and total T cell subpopulations (CD4^+^ and CD8^+^ T cells), inhibiting the adaptive immune responses in the murine liver. Indeed, it has been proven that T cell-modulated adaptive immune responses play an important role in CRC progression [53]. In this study, *F. nucleatum* exposure resulted in upregulation of Treg cell population in both the spleen and liver, which is further supported by the previous findings from Ma et al. [13]. A number of studies have demonstrated that Tregs play an important role in suppressing immune responses by modulating MDSCs to drive tolerance, suggesting that Tregs can potentially inhibit anti-tumor immunity in the liver [54, 55].

The balance between the Treg and Th17 cells is crucial for the regulation of pro- and anti-tumor immunity [56]. Elevated levels of Th17-related cytokines in advanced CRC are reported to be associated with poor outcomes [57]. In accordance with previous findings, the secretion of Th17-associated cytokines (TNF-α, IL6, and IL17A) was found to be significantly augmented in the plasma, concomitant with decreased infiltration of Th17 cells in the livers of mice belonging to the *F. nucleatum* group as compared to the PBS group. Th17 plays a dual role in CRC development [56]. Wang et al. have demonstrated an immunosuppressive effect of Th17 within the TME through inhibition of CD8^+^ T cell migration to tumors [58]. Nevertheless, previous studies have shown that Th17 cells can recruit effector T cells and NK cells to support anti-tumor immunity [59]. Ling et al. have confirmed that decreased Th17 infiltration is associated with CRC progression in patients [60], which is in agreement with our findings.

The role of the gut microbiota in modulating host immunity has received increasing attention in recent times. It is well-established that immune system influences the composition of the gut microbiota. Studies have demonstrated a critical role of the gut microbiota in the initiation and development of CRC, and that inflammation via IBD can drive the loss of microbiota diversity, leading to a distinct composition of microbial community [24, 61]. Our study has demonstrated that oral administration of *F. nucleatum* remarkably reduced the diversity of the intestinal microbiota and altered its composition as compared to the control group; although the *Fusobacteria* were not detected in the feces of mice, which may be attributed to the small group size and insufficient administration period. Further evaluation of the gut microbiota composition demonstrated a higher abundance of pathogenic bacteria, such as *Proteobacteria*, in the *F. nucleatum* group than in the PBS group at the phylum level. At the genus level, increased *Enterococcus* and *Escherichia/Shigella* abundance were detected in mice treated with *F. nucleatum* as compared to those administered with PBS as control. Consistent with our results, a reduction in the abundance of *Clostridia* and an elevated abundance of *Enterococcus* and *Escherichia/Shigella* has been reported in mice demonstrating CRLM [13]. Previous study thus highlights the driver-passenger model, wherein, the driver bacteria *Enterococcus* or *Escherichia/Shigella* produce genotoxic substances to damage the epithelial cell DNA, whereas *F. nucleatum* as the passenger bacterium favors the proliferation of cancer cells to promote CRC tumorigenesis [62]. In contrast, the administration of *F. nucleatum* was also found to be associated with decreased abundance of *Lachnospiraceae_NK4A136_group* and *Ruminiclostridium_9.* A previous study reported that *Lachnospiraceae_NK4A136_group* is more abundant in healthy individuals than in the CRC patients [63]. Furthermore, an elevated abundance of *Ruminiclostridium_9* has been documented post administration of a probiotic [64]. These results imply that *Lachnospiraceae_NK4A136_group* and *Ruminiclostridium_9* may play a protective role in CRC progression.

Despite significant studies, whether the alteration of gut microbiota is a reason or a consequence of liver metastasis of CRC is still unclear. Due to the gut-liver axis, the liver receives toxins and microorganisms via the portal vein from the gut to ensure that it can metabolize gut-derived microbial products and nutrients. Moreover, it has been documented that metastatic liver demonstrates altered hepatic cyto-architecture, ensuing a pro-inflammatory state and metabolic disorders [65, 66]. Accumulation of the harmful metabolic products damages the composition of healthy intestinal flora, altering the composition of gut microbiota. Moreover, the commensal bacteria are important regulators of anti-tumor immunity. It has been reported that alterations in the gut microbiota can exacerbate the liver metastases through the production of prostaglandins to suppress tumor immunity [67], increasing the intestinal permeability to induce the hepatic inflammation [68], and altering the bile acid composition [69]. In the present study, the administration of *F. nucleatum* was found to result in dysbiosis and modulated the hepatic immune environment to promote the liver metastases. Previous studies have shown that disequilibrium of intestinal microflora can regulate the liver immune environment through several mechanisms, including the activation of intestinal inflammation, and accumulation of bacterial as well as metabolic products [14, 70–72]. Certain metabolites secreted from *Enterococcus faecalis* or FadA in *F. nucleatum* have been reported to damage DNA, resulting in enhanced proliferation of colon cancer cells in studies on gut microbiota in cancer patients [73]. *Clostridium* and its metabolites have been shown to promote T cell accumulation and differentiation to alleviate colitis in mice [74]. *Enterococcus* and *Escherichia/Shigella* have been found to change gut permeability and promote inflammation in the liver because of the high level of lipopolysaccharides (LPS) [75]. Thus, *F. nucleatum* may partially promote CRLM through modulation of the liver immune microenvironment as a result of an imbalanced intestinal microflora. However, the exact mechanisms mandate further investigation to delineate the detailed molecular mechanism underlying the regulation of the liver immune response by the *F. nucleatum–*induced dysbiosis.

In conclusion, our study demonstrated that administration of *F. nucleatum* affects the secretion of inflammatory cytokines and modulates the hepatic immune response to promote CRLM. These changes are attributed to alterations in the structure and composition of the gut microbiota. However, these findings need to be validated with additional studies. Nonetheless, our findings provide new insights for the development of innovative therapeutic strategies for targeting microbiota in patients with CRLM.

## MATERIALS AND METHODS

### Bacterial culture

The *F. nucleatum* strain ATCC 25586 was purchased from the China General Microbiological Culture Collection Center (CGMCC, Beijing, China). The bacteria were grown in brain heart infusion (Beijing Land Bridge Technology, Beijing, China) broth medium supplemented with 5 mg/L heme (Beijing Land Bridge Technology) and 1 mg/L vitamin K1 (Beijing Land Bridge Technology) under anaerobic conditions (AnaeroPack; Mitsubishi Gas Chemical, Tokyo, Japan) at 37° C for 48 h before harvesting.

### Cell culture

The CT26 mouse colon carcinoma cell line was obtained from the Cell Bank of the Chinese Academy of Sciences (Shanghai, China) and stably transfected with the luciferase gene, to generate CT26-Luciferase (CT26-Luc) cell line. CT26-Luc colon cells were cultured in RPMI 1640 (HyClone, GE Healthcare; Chicago, IL, USA) supplemented with 10 % fetal bovine serum (FBS; Gibco, Thermo Fisher Scientific Corp., Waltham, MA, USA) at 37° C in a humidified 5 % CO_2_ atmosphere.

### Mice

Six to eight-week-old male BALB/c mice were purchased from the Experimental Animal Center of Hubei Province, China. All mice were raised under specific-pathogen-free barrier conditions and fed autoclaved food and water according to the institutional guidelines for Animal Care and Use Committee of Tongji Hospital, Huazhong University of Science and Technology, Hubei, China. The body weight of the mice was measured twice a week. All animal protocols were approved by the Ethical Committee on Animal Experiments.

Before intragastric administration of bacteria, streptomycin (2 mg/mL, Shanghai Macklin Biochemical Co., Ltd Shanghai, China) was administered to all mice used in the study via drinking water for three days. To investigate *F. nucleatum*-associated CRLM, 30 mice were randomly assigned to two groups and 10^9^ colony-forming units of *F. nucleatum* resuspended in PBS were administered to mice by gavage every day for eight weeks. After the 4-week *F. nucleatum* or PBS administration, the CT26-Luc cells were injected into the spleens of BALB/c mice to establish a liver metastasis model, which were denoted as *F. nucleatum* group and PBS group, respectively. Another 30 mice were administered with *F. nucleatum* or PBS until moribund to investigate *F. nucleatum*-associated survival.

### Flow cytometry

Livers and spleens were collected from mice post completion of the *in vivo* experiments. The tissues were minced and dissociated in cell culture medium containing 0.01 % type IV collagenase and 0.001 % DNase I for 45 min at 37° C. The mixture was subsequently filtered carefully through a 70-μm nylon cell strainer to obtain a single-cell suspension. The suspension was subsequently purified by gradient centrifugation with 30–70 % Percoll [76]. According to the manufacture’s recommendation, cell surface markers were stained with the indicated antibodies for 30 min at 4° C and live cells were surface-labeled by Fixable Viability Stain 780 (BD Biosciences, San Jose, CA, USA). Cells were then permeabilized in transcription factor buffer (BD Biosciences) and subjected to intracellular Foxp3 staining using relevant monoclonal antibodies. For intracellular IL17 staining, cells were stimulated in complete medium (RPMI 1640 containing 10 % FBS, 1 % penicillin and streptomycin) for 12 h in the presence of Leukocyte Activation Cocktail with GolgiPlug (2 μL/mL, BD Biosciences) and then stained with the indicated antibodies. The following antibodies were used for flow cytometry analysis: anti-CD8α-FITC (53-6.7, BD Biosciences), anti-IL17A-PE (TC11-18H10, BD Biosciences), anti-CD3-BB700 (145-2C11, BD Biosciences), anti-Foxp3 Alexa Fluor 647 (MF23, BD Biosciences), anti-CD4-BV421 (GK1.5, BD Biosciences), anti-CD45-BV480 (30-F11, BD Biosciences), anti-CD49b-PE (DX5, BD Biosciences), anti-CD206 Alexa Fluor 647 (MR5D3, BD Biosciences), anti-F4/80 BV421 (T45-2342, BD Biosciences), anti-CD11b-FITC (M1/70, BD Biosciences), and anti-Ly-6G/Ly-6C-PE-CF594 (RB6-8C5, BD Biosciences). Stained cells were collected using a CytoFLEX LX Flow Cytometer (Beckman Coulter, Carlsbad, CA, USA) and analyzed using FlowJo (FlowJo LLC, Ashland, OR, USA).

### Detection of plasma cytokines

Plasma cytokines were detected by Luminex suspension chip detection technology provided by Wayen Biotechnologies Inc., Shanghai, China. Following the manufacturer’s instructions, the Bio-Plex Pro Mouse Cytokine Array kit was used for detection of cytokines. Diluted mouse plasma was incubated in a 96-well plate embedded with microbeads for 30 min at room temperature under dark conditions. The samples were then incubated with the detection antibody for 30 min. Subsequently, streptavidin-PE was added to each well for the development of color rendering, and the calibrated Luminex 200 machine was used to measure its value.

### Establishment of liver metastasis model and its imaging

The liver metastasis model was generated by intra-splenic injection of 1 × 10^6^ CT26-Luc cells via an insulin syringe, and the compression of blood after 3 min of hemostasis. After four weeks, fluorescent images were captured using *in vivo* luciferase imaging system to observe the metastatic foci in the liver. All mice were anesthetized with 1.5 % pentobarbital sodium and euthanized for tumor statistics and histopathologic analysis. The livers and cecum were also dissected, weighed, and photographed.

The development of metastases was monitored by IVIS (Lago, Spectral Instruments Imaging; Tucson, AZ, USA). All mice were anesthetized with 1.5 % pentobarbital sodium and injected intraperitoneally with D-luciferin at a dose of 1.5 mg per 10 g body weight, and 15 min later, luciferase imaging was performed. The images were analyzed using Living Image software (PerkinElmer, Waltham, MA, USA). An equal size of the abdominal region was assigned as the ROI, and the number of photons emitted per second (photon/s) in the ROI was acquired for quantitative analysis.

### High-throughput sequencing

Stool samples from the distal rectum were immediately collected and stored in liquid nitrogen after euthanizing the mice. The concentration of DNA extracted from the stool was detected using a Qubit 2.0 Fluorometer (Invitrogen, Thermo Fisher Scientific Corp., Waltham, MA, USA). High-throughput sequencing and construction of the sequencing library were performed using Illumina MiSeq (Illumina, San Diego, CA, USA) platform as per the manufacturer’s instructions. Operational taxonomic unit analysis clustered sequences at 97 % similarity level using VSEARCH (version 1.9.6) [77]. The rRNA gene database Silva132 was selected for species annotation and taxonomic analysis based on the Ribosomal Database Program (RDP) classifier algorithm [78]. Alpha diversity was evaluated using the Shannon and Chao1 index (http://www.mothur.org/wiki). Beta diversity analysis was performed using the Bray-Curtis algorithm and (un)weighted UniFrac. PCoA and NMDS analysis were performed, and rank-abundance and rarefaction curves were plotted using QIIME (version 1.9.1) and R software [79]. The LEfSe was analyzed using online tools (http://huttenhower.sph.harvard.edu/galaxy/root?tool_id=lefse_upload), with an LDA score of 3.5 as the cut-off threshold for significant differences. The data were submitted to the Sequence Read Archive database (PRJNA676655).

### Hematoxylin and eosin (H&E) staining

The liver tissue with or without metastasis in each group was fixed in 4 % paraformaldehyde (Wuhan Servicebio Technology Co., Ltd., Hubei, China) and embedded in paraffin. Paraffin sections (5 μm) were then stained with H&E (Biossci, Hubei, China) to determine hepatic metastases.

### Statistical analysis

Data are presented as the mean ± standard deviation or the mean ± standard error of the mean and analyzed using GraphPad Prism 6.0 (GraphPad Software, CA, USA). Survival analysis was assessed using the Kaplan-Meier method, and the log-rank test was used to compare the difference in survival of the two groups. Student’s t-test, the Mann-Whitney U test, and one-way analysis of variance were used for statistical analysis of the data. Differences were considered statistically significant at *P*< 0.05.
